# *Moringa peregrina* Leaves Extracts Induce Apoptosis and Cell Cycle Arrest of Hepatocellular Carcinoma

**DOI:** 10.1155/2019/2698570

**Published:** 2019-01-01

**Authors:** Mohamed Mansour, Magda F. Mohamed, Abeer Elhalwagi, Hanaiya A. El-Itriby, Hossam H. Shawki, Ismail A. Abdelhamid

**Affiliations:** ^1^Department of Chemistry, Faculty of Science, Cairo University, 12613 Giza, Egypt; ^2^National Gene Bank of Egypt (NGB), Agricultural Research Center (ARC), Giza, Egypt; ^3^Department of Chemistry (Biochemistry Branch), Faculty of Science, Cairo University, Giza, Egypt; ^4^Department of Comparative and Experimental Medicine, Nagoya City University Graduate School of Medical Sciences, Nagoya, Japan

## Abstract

Moringa grows in the tropical and subtropical regions of the world. The genus* Moringa* belongs to family Moringaceae. It is found to possess various medicinal uses including hypoglycemic, analgesic, anti-inflammatory, hypolipidemic, and antioxidant activities. In this study, we investigated the antimicrobial and the anticancer activity of the* Moringa peregrina* as well as* Moringa oleifera* leaves extracts grown locally in Egypt. Results indicated that most of the extracts were found to possess high antimicrobial activity against gram-positive bacteria, gram-negative bacteria, and fungus. The survival rate of cancer cells was decreased in both hepatocellular carcinoma (HepG2) and breast carcinoma (MCF-7) cell lines when treated with* Moringa* leaves extracts. In addition, the cell cycle progression, apoptosis, and cancer-related genes confirmed its anticancer effect. The toxicity of each extract was also tested using the normal melanocytes cell line HFB4. The toxicity was low in both* Moringa peregrina* and* Moringa oleifera* leaves extracts. Furthermore, GC/MS analysis fractionized the phytochemicals content for each potential extract. In conclusion, results suggested that the* Moringa peregrina* and* Moringa oleifera *leaves extracts possess antimicrobial and anticancer properties which could be attributed to the bioactive phytochemical compounds present inside the extracts from this plant. These findings can be used to develop new drugs, especially for liver cancer chemotherapy.

## 1. Introduction

One of the richest sources of natural bioactive phytochemicals is the plant kingdom. The uses of plants in medicine are very old thought [1]. The lower toxicity and side effects of bioactive phytochemicals than the synthetic drugs made the uses of medicinal plants in treatment more desirable [2]. In addition, the presence of multiple phytochemicals molecules in a plant supports the participation in complex cellular pathways [1].

*Moringa peregrina* and* Moringa oleifera* belong to the family of Moringaceae. Moringa is a perennial and fast-growing tree that could reach a 7-12 m of maximum height with 20-40 cm of diameter with respect to chest level and grows naturally at up to 1000 m above sea level [3]. Moringa genus has 13 species spread around northeast Africa, southwest Africa, southwest Asia, and Madagascar [4]. Egyptians had used* Moringa* trees since old and middle kingdoms (3000-2000 B.C.) [5]. It was used traditionally to improve the overall body health. In addition, during wars the* Moringa oleifera* leaves were used by the Indian warriors to enhance their energy and reduce pain and stress [6].

*Moringa* trees possess high nutritional value because of the numerous essential phytochemical compounds presented in all its parts (leaves, pods, and/or seeds). Previous studies stated that* Moringa* leaves have vitamin C content more than oranges by 7 times, protein content more than yoghurt by 9 times, vitamin A content more than carrots by 10 times, potassium content more than bananas by 15 times, calcium content more than milk by 17 times, and iron content more than spinach by 25 times [1, 7]. Many studies on* Moringa oleifera* tree have discovered promising anticancer [8], anti-inflammatory [9], procoagulant [10], water purification [11], antifungal [12], and antibacterial [13] properties.

In this study, we focused on two* Moringa* species grown in Egypt (*Moring peregrina *and* Moringa oleifera*) to examine the effect of their leaves serial-extraction as an antimicrobial agent against gram-positive bacteria, gram-negative bacteria, and fungus as well as an anticancer agent against hepatocellular carcinoma (HepG2) and breast carcinoma (MCF-7) cell lines. In addition, cell survival, apoptosis, cell cycle progression, and cancer-related gene were examined to confirm the anticancer effect. Moreover, GC/MS analysis for each serial extract was done to rationalize this activity according to its leave extracts phytochemicals content.

## 2. Materials and Methods

### 2.1. Collection of Plants

*Moringa peregrina* and* Moringa oleifera *leaves were collected from trees in the Orman Garden, Ministry of Agriculture, Egypt. 2 kg of fresh leaves were collected for each species and air-dried in a shaded area, and the leaves were grinded into a coarse powder using a laboratory grinder.

### 2.2. Preparation of the Extracts

Serial extracts for* Moringa peregrina* and* Moringa oleifera* leaves were done using 5 solvents differing in polarity: Hexane, Diethyl ether, Ethyl acetate, Methanol, and Acetonitrile in series. The abbreviations used for each extract are explained in Table S1. Each of the coarsely powdered specimens was taken into a round bottom flask and 1000 ml of the hexane was added. The soluble constituents of the extract were dissolved in the solvent by overnight shaking. The soluble extracts were filtered and evaporated in a rotary evaporator (IKA, Germany; temp: 50°C; pressure 175 mbar) to yield semisolid residue. Supplemental Fig S1 shows the full extraction scheme. The remaining plant tissue from the hexane extraction was kept till using with the next solvent. The same is done until we finished the other 4 extraction solvents. Finally, the extract residues were collected and stored at 4°C until further uses.

### 2.3. Assay of Antimicrobial Activity

The antimicrobial activity of each* Moringa peregrina* and* Moringa oleifera* leaves extracts was determined using the agar well diffusion method [14]. Each extract was tested in vitro for its antibacterial activity against gram-positive bacteria (*Staphylococcus aureus* and* Streptococcus mutans*) and gram-negative bacteria (*Escherichia coli*,* Pseudomonas aeruginosa,* and* Klebsiella pneumonia*) and for its antifungal activity against* Candida albicans* using nutrient agar medium. Ampicillin, Gentamicin, and Nystatin were used as standard drugs for gram-positive and gram-negative bacteria and fungus, respectively. DMSO was used as the solvent control. The test was done at a concentration of 15 mg/ml from each extract against both bacterial and fungal strains. The sterilized media was poured onto the sterilized Petri dishes (20-25 ml, each petri dish) and allowed to solidify at room temperature. The microbial suspension was prepared in sterilized saline equivalent to McFarland 0.5 standard solution (1.5 x 10^5^ CFU mL^−1^) and its turbidity was adjusted to OD = 0.13 using spectrophotometer at 625 nm. Optimally, within 15 minutes after adjusting the turbidity of the inoculum suspension, a sterile cotton swab was dipped into the adjusted suspension and was flooded on the dried agar surface and then allowed to dry for 15 min with lid in place. Wells of 6 mm diameter were made in the solidified media with the help of sterile borer. 100 *μ*L of the solution of each extract was added to each well with the help of micropipette. The plates were incubated at 37°C for 24 h. This experiment was carried out in triplicate and zones of inhibition were measured in mm scale.

### 2.4. Single Dose Measurement of the Cytotoxicity against Cell Lines Using SRB Assay

Potential of cytotoxicity of the 5 extract residues form each species was tested against two cancer cell lines (HepG2 and MCF-7) and the normal melanocytes cell line HFB4 using SRB assay method [15]. Each cell line was plated into 96-multiwell plate (10^4^ cells/well) for 24 h before treatment to allow attachment of the cells to the plate wall. Then, a single dose of each extract (20 *µ*g/ml) was added to each cell line. Monolayer triplicate wells were prepared for each individual dose. Monolayers cells were incubated with the extracts for 48 h at 37°C in an atmosphere of 5% CO_2_. After 48 h, cells were fixed, washed, and stained with Sulfo-Rhodamine-B stain. Excess stain was washed with acetic acid and attached stain was recovered with Tris EDTA buffer. The color intensity was measured using an ELISA reader. Doxorubicin was used as a positive control for the HFB4 cell line.

### 2.5. MTT Assay

MTT assay is a sensitive, quantitative, and reliable colorimetric method that measures the viability of cells. The assay is based on the ability of mitochondrial lactate dehydrogenase enzymes (LDH) in living cells to convert the water-soluble substrate 3-(4,5-dimethylthiazol-2-yl)2,5diphenyl tetrazolium bromide (MTT) into a dark blue formazan which is water insoluble. A solubilization solution (dimethyl sulfoxide) is added to dissolve the insoluble purple formazan product into a colored solution. The absorbance of this colored solution can be quantified by measuring it using spectrophotometer at a wavelength usually between 500 and 600 nm [16]. The assay modification was done according to our previous work [17–19]. Different concentrations of each extract (0.5, 1, 2, 4, 6, 8, 16, 32, 62, 125, 250, 500, and 1000 mg/ml) were incubated with the HepG2 cell line. After 48 h of incubation at 37°C, the cells were incubated for 4 h at 37°C with MTT (0.8 mg/ml) and dissolved in serum-free mediums. Then the MTT was discarded and the cells were washed three times using 1 ml of PBS, followed by the addition of 1ml of DMSO. Then gentle shaking for 10 min was done until complete dissolution. 200 *μ*l of the resulting solutions for each extract was transferred to 96-well plates. The optical densities (ODs) were measured at 570 nm using an ELISA plate reader. Viability percentage was calculated as follows: cell viability percentage = (OD of treated cells/OD of untreated cells) X 100. IC_50_ of the 4 extract residues was measured using Prism program (Graphpad Software incorporated, version 3).

### 2.6. Quantitative RT-PCR

The expression of* BAX*,* BCL2*,* P53*,* CASP3*, and* MMP1* genes was examined according to Ali et al. [20]. Total RNA was isolated from HepG2 cells treated with a concentration equal to the IC_50_ values of the most active extracts (O/DEE, O/EA, P/DEE, and P/EA). The RNA from untreated HepG2 cells was used as a control. The isolation and purification were done using Qiagen RNA extraction kit. The purity and the yield of extracted RNA were tested at 260 nm. SIGMA PCR kit was used for the synthesis of the cDNA strands and the real-time PCR test was done in a single tube using Rotor gene PCR system as a reader. The primers sequence for the tested genes (*BAX*,* BCL2*,* P53*,* CASP3,* and* MMP1*) and the reference housekeeping gene* GAPDH* are shown in Table S2. The recorded cycle threshold (Ct) values of the targeted genes were used to calculate the relative quantitation (RQ) by calculating the delta-delta Ct (ΔΔCt).

### 2.7. Flow Cytometry

The method was carried out as previously described [21]. Cell cycle distribution analysis by quantitative DNA content of HepG2 cells treated with the IC_50_ concentration of the Moringa extracts was performed using Propidium Iodide (PI) Flow Cytometry Kit for Cell Cycle Analysis (Abcam, Cat. # ab139418). The untreated HepG2 cells were used as a control. Briefly, cells were prepared at a density of 1x10^4^ per well, treated with the extracts for 24 h at 37°C, harvested in a single cell suspension, and fixed with 66% ethanol at 4°C. Cells were then stained by PI and cycle distribution was determined by using the FACS Calibur (BD Biosciences, San Jose, CA, USA). The analysis was done using BD CellQuest™ Pro Analysis software (BD Biosciences, San Jose, CA, USA). The percentage of apoptosis was recorded.

### 2.8. Gas Chromatography

The gas chromatographic analysis was carried out for the* Moringa* extracts using GC (Agilent Technologies 7890A) interfaced with a mass selective detector (MSD, Agilent 7000) equipped with a nonpolar Agilent HP-5ms ((5%-phenyl)-methylpolysiloxane) capillary column (30 m length X 0.25 mm inner diameter and 0.25 *µ*m film thickness). The carrier gas was helium with the linear velocity of 1 ml/min. The injector and detector temperatures were 200 and 250°C, respectively. A volume of 1 *µ*l of each extract was injected. The MS operating parameters were as follows: ionization potential 70 eV, interface temperature 250°C, and acquisition mass range 50-800 m/z. The identification of components was based on the comparison of their mass spectra and retention time with those of the authentic compounds and by computer matching with NIST and WILEY library as well as the comparison of the fragmentation pattern of the mass spectra data with those reported in the literature.

## 3. Results

### 3.1. Antimicrobial Activity of the* Moringa* Extracts

**Figure 1:**
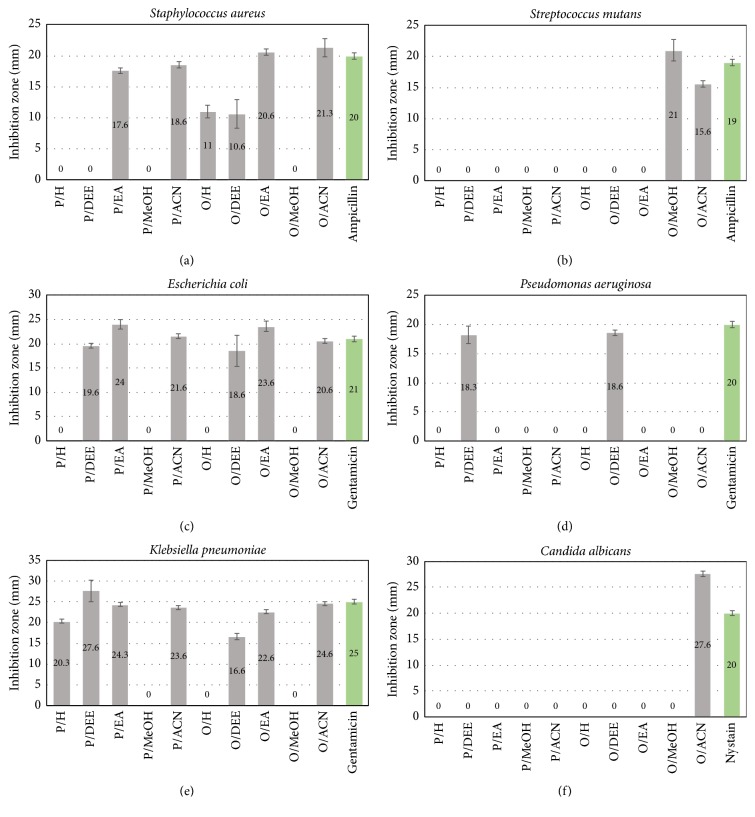
**The antimicrobial activity of the* Moringa peregrina* and* Moringa oleifera *leaves extracts**. P/H, P/DEE, P/EA, P/MeOH, P/ACN, O/H, O/DEE, O/EA, O/MeOH, and O/ACN extracts were tested for their inhibition effect on (a)* Staphylococcus aureus,* (b)* Streptococcus mutans, *(c)* Escherichia coli,* (d)* Pseudomonas aeruginosa,* (e)* Klebsiella pneumonia,* and (f)* Candida albicans.* Ampicillin antibiotic was used as the positive control for gram-positive bacteria, Gentamicin for gram-negative bacteria, and Nystatin for fungus. Numbers above columns indicate the inhibition zones (mm).

The antibacterial and antifungal activity of the* Moringa* extracts have been investigated using agar well diffusion method as explained in Materials and Methods. Each extract was tested for its antibacterial activity against gram-positive bacteria (*Staphylococcus aureus* and* Streptococcus mutans*) and gram-negative bacteria (*Escherichia coli*,* Pseudomonas aeruginosa,* and* Klebsiella pneumonia*) and for its anti-fungal activity against* Candida albicans *(Figure 1). Results in Figures 1(a) and 1(b) reveal that the extracts of P/EA, P/ACN, O/H, O/DEE, and O/EA had the ability to inhibit* Staphylococcus aureus*, while O/MeOH extract had the ability to inhibit the growth of* Streptococcus mutans *as compared to the positive control (Ampicillin). Moreover, O/ACN extract had the ability to inhibit growth of both the tested gram-positive bacteria:* Staphylococcus aureus* and* Streptococcus mutans*. Results of testing the extracts against gram-negative bacteria in Figures 1(c)–1(e) reveal that P/EA, P/ACN, O/EA, and O/ACN extracts had the ability to inhibit* Escherichia coli*, while P/H, P/EA, P/ACN, O/EA, and O/ACN extracts had the ability to inhibit the growth of* Klebsiella pneumoniae *as compared to the positive control (Gentamicin). Moreover, the diethyl ether extracts (O/DEE and P/DEE) had the ability to inhibit growth of the all tested gram-negative bacteria:* Escherichia coli*,* Pseudomonas aeruginosa, and Klebsiella pneumoniae*. On the other hand, extracts tested for their antifungal activity against* Candida albicans* in Figure 1(f) reveal that O/ACN was the only powerful extract effectively inhibiting the fungus with a high zone of inhibition 27.6 mm as compared to the positive control (Nystatin) which was 20 mm.

### 3.2. The Potential Cytotoxicity against Hepatocellular and Breast Carcinoma

Single dose cytotoxicity test was performed using sulforhodamine-B (SRB) assay to screen the anticancer activity of the* Moringa* leaves extracts.  Table 1 shows the inhibition effect of all extracts against HepG2 and MCF-7 cancer cell lines. The toxicity of the extracts against the normal melanocytes cell line HFB4 was done as shown in Table 2.* Moringa peregrina* and* Moringa oleifera *leaves ethyl acetate extracts (P/EA and O/EA) exhibited the highest inhibition activity against hepatocellular carcinoma HepG2 cell line (78% and 80.7% inhibition, respectively). In the same time, P/EA and O/EA show very low toxicity effect against the normal melanocytes cell line HFB4 (75% and 80% survival, respectively).* Moringa peregrina* and* Moringa oleifera *leaves diethyl ether extracts (P/DEE and O/DEE) were noted to be the most active extracts against breast carcinoma MCF-7 cell line with 79.4% and 80.3% inhibition, respectively. Both of P/DEE and O/DEE recorded the lowest toxicity effect on HFB4 cell line (87% survival) in comparison to the positive control Doxorubicin (21% survival). The rest of the extracts showed a moderate to a high response regarding their activity against HepG2 and MCF-7 cell lines. P/DEE, P/EA, O/DEE, and O/EA were the most selective and promising extracts with high anticancer activity and low toxicity.

### 3.3. IC50 Determination for Cell Inhibition

**Figure 2:**
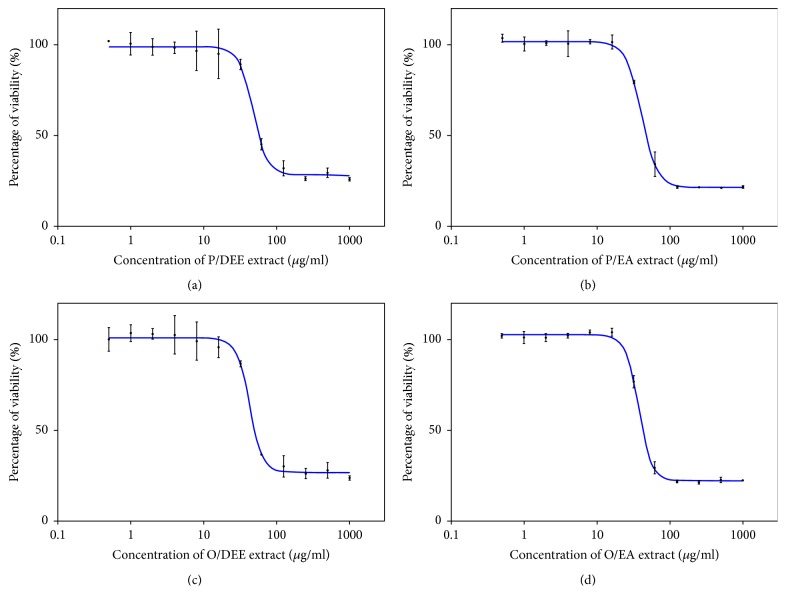
**Cytotoxicity evaluations of the 4 most active* Moringa* leaves extracts against HepG2 cell line after 48 h of treatment**. (a) P/DEE extract. (b) P/EA extract. (c) O/DEE extract. (d) O/EA extract. The IC_50_ values were calculated using Prism software program (GraphPad software incorporated, version 3). The blue curve indicates the nonlinear regression.

From the results above the most active extract residues were P/DEE, P/EA, O/DEE, and O/EA. Cytotoxicity against a model of study, HepG2 cell line, of the 4 most active* Moringa* leaves extracts was tested using MTT assay to find the IC_50_ values (concentrations that inhibited 50% of cell proliferation) of each (Figure 2). All of the 4 extracts exhibited high activity with low IC_50_ values relative to the positive control 5-fluorouracil (5-FU; IC_50_ 237 ± 1.153 *µ*g/ml). The O/EA was the most active extract with lowest IC_50_ value (37.23 ± 0.645 *µ*g/ml), followed by P/EA and O/DEE which recorded IC_50_ values of 40.72 ± 1.060 *µ*g/ml and 42.56 ± 1.060 *µ*g/ml, respectively. P/DEE lied at the end with IC_50_ value of 47.76 ± 2.485 *µ*g/ml.

### 3.4. The Expression Level of Cancer-Related Genes

**Figure 3:**
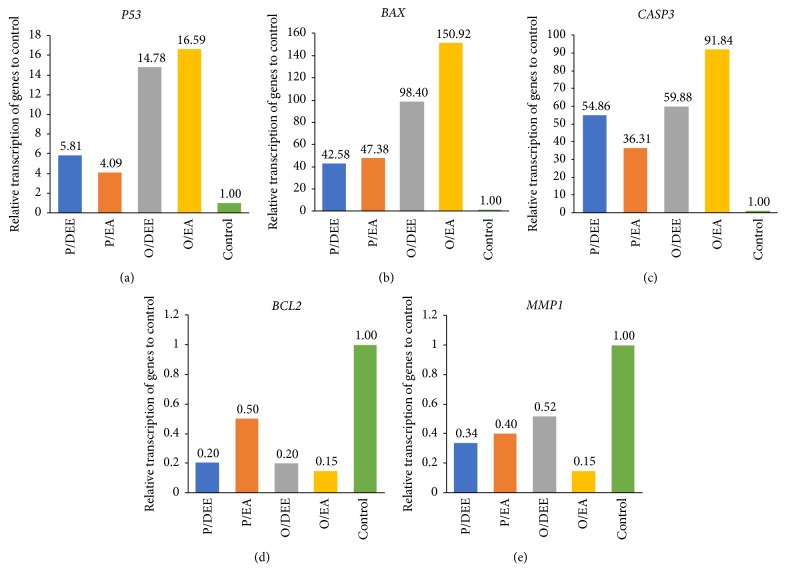
**The effect of* Moringa* leaves extracts on HepG2 mRNA transcription**. The relative transcription level of 5 cancer-related genes: (a)* P53,* (b)* BAX, *(c)* CASP3*, (d)* BCL2,* and (e)* MMP1 *were determined by qRT-PCR from HepG2 cells treated for 48 h. Values above columns indicate the fold change compared to control. Gene expression levels were normalized to* GAPDH*.

The 4 leaves extracts, P/DEE, P/EA, O/DEE, and O/EA, were selected for the molecular studies against hepatocellular carcinoma HepG2 as they exhibited best cytotoxicity and selectivity. The treated HepG2 cells and the untreated (control) were collected for genes expression analysis of the 5 following genes:* P53*,* BAX*,* CASP3*,* BCL2,* and* MMP1*. As shown in Figure 3(a), the 4 extracts induce the expression of the tumor suppressor gene* P53*. O/EA extract had the highest induction effect (17.59-fold) followed by O/DEE and P/DEE (14.78- and 5.81-fold, respectively). The lowest induction was P/EA (4.09-fold). The proapoptotic protein BAX (Figure 3(b)): O/EA had the highest effect on the expression level of* BAX* gene (150.992-fold increase) followed by O/DEE, P/EA, and P/DEE (98.40-, 47.38-, and 42.58-fold, respectively). At the same trend, the expression level of* CASP3 *gene (an inducer of the execution phase of cell apoptosis) was highly increased by the treatment of the 4 extracts (Figure 3(c)). The fold change was 91.84-, 59.88-, 54.86-, and 36.31-fold for O/EA, O/DEE, P/DEE, and P/EA, respectively. On the other hand, the expression level of two antiapoptotic genes,* BCL2* and* MMP1*, was strongly decreased in HepG2 cells upon treatment with the 4 extracts (Figures 3(d) and 3(e)). As shown in Figure 3(d), the O/EA had the highest decrease of* BCL2* followed by P/DEE, O/DEE, and P/EA. Similarly, Figure 3(e) shows that O/EA had the highest decrease of* MMP1* followed by O/DEE, P/DEE, and P/EA.

### 3.5. Extract Effects on Cell Cycle Arrest and Apoptosis

**Figure 4:**
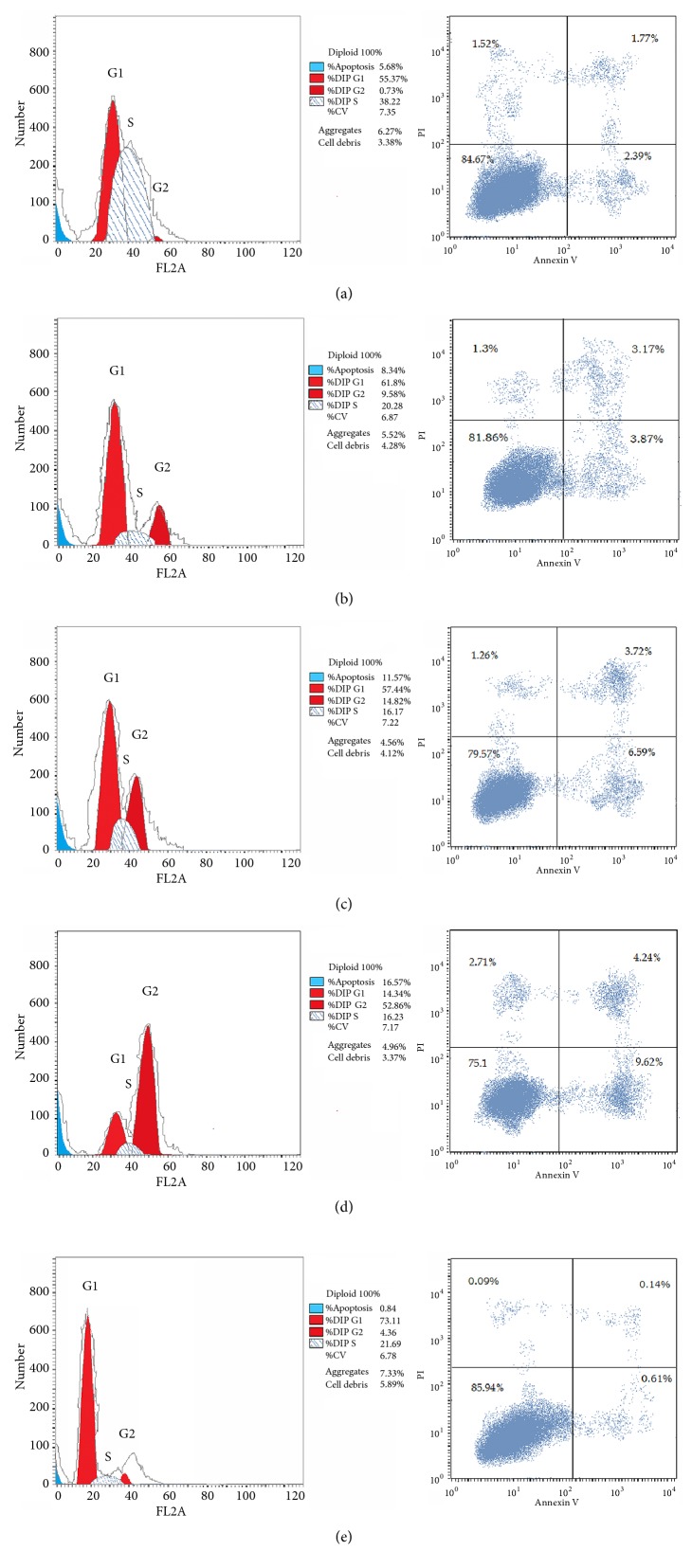
***Moringa* leaves extracts induce cell cycle arrest and apoptosis in HepG2 cells**. Cells treated for 48 hours with 4* Moringa* leaves extracts including (a) P/DEE, (b) P/EA, (c) O/DEE, (d) O/EA, and (e) untreated cells as controls. The cell cycle distribution was determined by propidium iodide staining (PI) and flow cytometry. Left panels show the distribution and the percentage of cells in phases of the cell cycle. Right panels show the distribution and the percentage of cells of apoptotic cells (Annexin^+^). The cell cycle phases G1, S, and G2/M are indicated over the peaks. PI: cell survival marker; Annexin V: apoptotic marker.

The effects of the 4* Moringa* leaves extracts, P/DEE, P/EA, O/DEE, and O/EA, on HepG2 cell cycle progression and apoptosis were examined. Results indicated that the P/DEE extract induced cell cycle arrest at S phase (Table 3 and Figure 4(a)), while cells treated with P/EA, O/DEE, and O/EA extracts were arrested at G2/M phase as shown in Table 3 and Figures 4(b), 4(c), and 4(d). Annexin V was used to detect the apoptotic cells of HepG2 after treatment with the different extracts. Results revealed that the percentage of apoptotic HepG2 cells increased with the stimulating effect of the 4 extracts as compared with the control (Table 3 and Figure 4). The O/EA extract stimulated the highest apoptotic induction effect 16.57 % followed by O/DEE, P/EA, and P/DEE (11.57%, 8.34%, and 5.68%, respectively), while the control group was 0.84 %.

### 3.6. Identification of Bioactive Compounds of Each Extract

**Figure 5:**
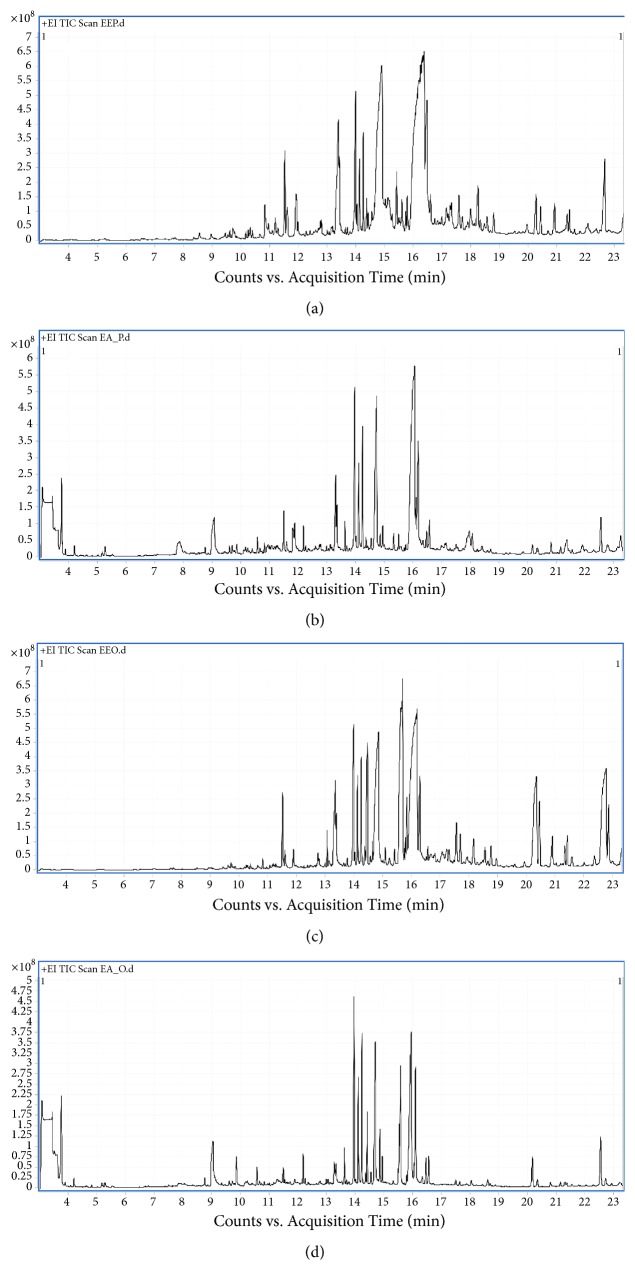
**GC/MS chromatograms of the active* Moringa* leaves extracts**. The chromatograms of (a) P/DEE, (b) P/EA, (c) O/DEE, and (d) O/EA extracts were analyzed using a GC (Agilent Technologies 7890A) interfaced with a mass selective detector (MSD, Agilent 7000) equipped with a nonpolar Agilent HP-5ms ((5%-phenyl)-methylpolysiloxane) capillary column. The carrier gas was helium with a linear velocity of 1 ml/min. The injector and detector temperatures were 200 and 250°C, respectively. A volume of 1 *µ*l of each extract was injected. The MS operating parameters were as follows: Ionization potential 70 eV, interface temperature 250°C, and acquisition mass range 50-800 m/z.

**Figure 6:**
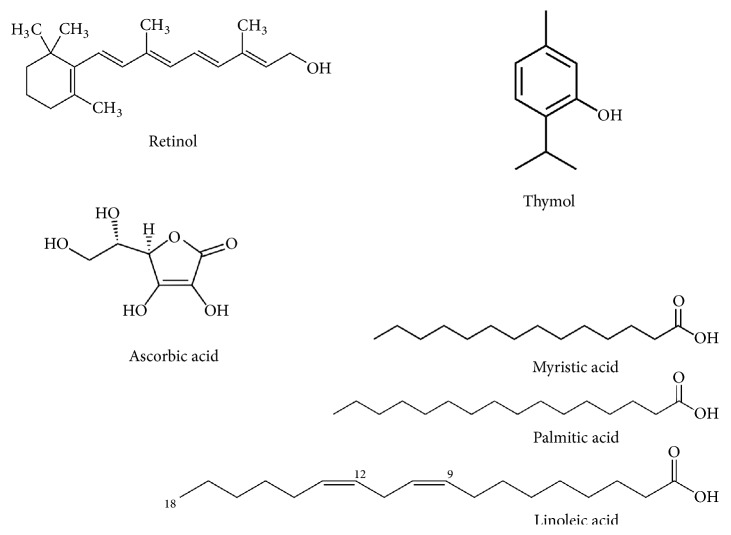
**Chemical structure of the bioactive compounds from* Moringa* extracts**. The major bioactive compounds were separated and identified from the 4* Moringa* leaves extracts using the GC/MS. The name of each compound is indicated.

GC/MS analyses of the 4 most active* Moringa* leaves extracts (P/DEE, P/EA, O/DEE, and O/EA) were done to identify any bioactive phytochemical compound. P/DEE showed 18 peaks on chromatogram, representing the phytochemical compound within this extract (Figure 5(a)). P/EA, O/DEE, and O/EA extracts showed 23, 34, and 14 peaks, respectively (Figures 5(b)–5(d)). The separated compounds from the 4 extracts were grouped as phenolics, hydrocarbons, long chain fatty acids, alcohols, and esters as summarized in Table 4. Several bioactive compounds were recognized in the leaves extracts of* Moringa peregrine* and* Moringa oleifera. *The chemical structures of some distinguished bioactive compounds identified and reported in literature including retinol, thymol, ascorbic acid, myristic acid, palmitic acid, and linoleic acid are illustrated in Figure 6.

## 4. Discussion

Over the last few years, plant phytochemicals, especially the ones possessing anticancer activity, gained extensive attention [22].* Moringa* trees are one of the plants that have been adapted in several tropical and subtropical regions of the world [7]. Many bioactive phytochemical compounds with a high medicinal and nutritional value were reported for this plant [23]. Previous studies confirmed that* Moringa oleifera* leaves possess anticancer [8, 24], anti-inflammatory [9], procoagulant [10], antifungal [12], and antibacterial [13] properties. However, the medicinal properties for* Moringa peregrine *leaves were not well examined yet. In the current study, we used five serial extracts from* Moring peregrine *as well as* Moringa oleifera leaves* grown in Egypt, to examine their effectiveness as antimicrobial agent and anticancer agent against hepatocellular carcinoma (HepG2) and breast carcinoma (MCF-7) cell lines.

*Moringa oleifera* leaves were reported to possess antimicrobial activity against* Pseudomonas aeruginosa* and* Staphylococcus aureus* but not other gram-positive and gram-negative bacteria and fungus [25]. Our results indicated that leave extracts from* Moring peregrine* as well as* Moringa oleifera* locally growing in Egypt had antimicrobial activity against gram-positive and gram-negative bacteria and fungus. However, although most of the serial extracts have the antimicrobial activity, each of them has a specific activity against specific types of bacteria. We found that O/ACN was the most powerful extract to inhibit the growth of gram-positive bacteria, while O/DEE and P/DEE extracts were the most powerful to inhibit gram-negative bacteria. In addition, O/ACN was the only powerful extract effectively inhibiting the fungus. Therefore, these results could be attributed to the different bioactive compounds present within each extract.

The serial extracts of* Moringa peregrina* and* Moringa oleifera* leaves were then examined for their anticancer activity. The results revealed that* Moringa peregrine *leaves had anticancer activity the same as that previously reported for* Moringa oleifera *[24]. The bioactive compounds from the ethyl acetate extracts of both* Moringa* species (P/EA and O/EA) exhibited the highest inhibitory effect against hepatocellular carcinoma, while diethyl ether extracts (P/DEE and O/DEE) exhibited the highest inhibitory effect against breast carcinoma. Notably, these effects came from the extracts but not the solvent DMSO which were reported to have no biological effects at the final concentration of 0.1% [17, 26, 27]. Moreover, the active extracts (P/DEE, P/EA, O/DEE, and O/EA) had low toxicity effect against the normal melanocytes cell and low IC_50_ values. The effects of these four active extracts, P/DEE, P/EA, O/DEE, and O/EA, as an anticancer agent were confirmed by the expression level of cancer-related genes. Previous studies indicated that* p53* is a tumor suppressive [28],* BAX* is an apoptotic cell death inducer [29], and* CASP3* is a crucial mediator of apoptosis [30], while* BCL-2* is an antiapoptotic gene [31] and* MMP1* inducer of cancer cell proliferation [32]. Our data revealed that HepG2 cells treated with any of these 4 active extracts promote cell apoptosis by upregulation of* p53*,* BAX*, and* CASP3* and downregulation of* BCL-2* and* MMP1*. In this connection,* p53* is known to trigger apoptosis and cell cycle arrest at S phase and G2/M phase [33–36]. We similarly found that P/DEE extract induces cell arrest at S phase, along with P/EA, O/DEE, and O/EA extracts that induce cell cycle arrest at G2/M phase. Transcription factor p53 binds to the DNA-binding domain of the antiapoptotic BCL-2 protein which disrupts the BCL-2/BAX complex and that promotes the permeabilization of the mitochondrial membrane [37, 38]. Consequently, mitochondria permeabilization leads to activation of the caspase cascades and results in cell cycle arrest and apoptotic cell death. These events were totally matched with the observation of our gene expression analyses.

GC/MS of the 4 active* Moringa* leaves extracts were examined to detect bioactive compounds. Previously, it has been described that plants containing high phenolic contents have a considerable anticancer activity and are counted as anticancer potential source [39–42]. Moreover, extracts with long chain fatty acids and their derivatives are also considered as anticancer sources [43]. Our results were detected within the leaves extract: phenolic compounds (thymol and ascorbic acid), long chain fatty acids (myristic acid, palmitic acid, and linoleic acid), and retinol which is known as a cancer treatment [44]. Future studies are required to separate the bioactive compound from the leaves of* Moringa peregrina* and* Moringa oleifera* in order to identify the exact anticancer compounds. These results will contribute to developing anticancer drug for hepatocellular carcinoma from natural compounds.

## 5. Conclusions

The serial leaves extract of* Moringa peregrina* as well as* Moringa oleifera* exhibited antimicrobial effects against gram-positive bacteria, gram-negative bacteria, and fungus. Extracts also exhibited cytotoxic effect against HepG2 and MCF-7 cell lines while exhibiting low toxicity on the normal melanocytes cell line. Diethyl ether and ethyl acetate extract methods were highly effective for anticancer activity by inducing cell cycle arrest and apoptosis of the HepG2 cells. GC/MS analysis showed that diethyl ether and ethyl acetate leaves extracts were rich in retinol, thymol, ascorbic acid, myristic acid, palmitic acid, and linoleic acid that would explain this activity.

## Figures and Tables

**Table 1:** The inhibition percentage of HepG2 and MCF-7 cell lines by each extract used.

*M. Peregrina extracts*	% inhibition		*M. Oleifera extracts*	% inhibition	
	HepG2	MCF7		HepG2	MCF7
P/H	62.8	73.6	O/H	73.5	61.3
P/DEE	77.3	79.4	O/DEE	72.2	80.3
P/EA	78	65.7	O/EA	80.7	63.5
P/MeOH	69	61.3	O/MeOH	76.7	61.3
P/ACN	76.7	59.7	O/ACN	79.7	52.8

**Table 2:** The survival percentage of HFB4 cell line incubated with each extract for 48 h.

Treatment	% survival	Treatment	% survival	Treatment	% survival
*M. Peregrina *		*M. Oleifera*		*Positive control*	
P/H	80	O/H	83	Doxorubicin	21
P/DEE	87	O/DEE	87		
P/EA	75	O/EA	80		
P/MeOH	77	O/MeOH	80		
P/ACN	81	O/ACN	82		

**Table 3:** Cell cycle phases and apoptosis of each extract compared to its control.

**Sample Code**	%**G0-G1**	%**S**	%**G2-M**	%**Apoptosis**
**P/DEE**	55.37	38.22	0.73	5.68
**P/EA**	61.8	20.28	9.58	8.34
**O/DEE**	57.44	16.17	14.82	11.57
**O/EA**	14.34	16.23	52.86	16.57
**Control HepG2**	73.11	21.69	4.36	0.84

**Table 4:** Compounds found in each extract using the GC/MS analysis.

Extract	(RT min) Compounds names
P/DEE	(9.065) Sorbitol, (10.809) Hexamethylbenzene, (11.502) Butylated Hydroxytoluene, (11.9) Oleic Acid, (13.374) 6-tert-Butyl-2,4-dimethylphenol, (13.971) Palmitaldehyde, (14.116) Levomenthol, (14.219) Isophytol, (14.83) Isopropyl palmitate, (16.084) Isopropyl linoleate, (16.225) Tetracosanoic acid, (17.668) Methyl tridecanoate, (18.245) Arachic acid, (20.25) Hexacosane, (20.865) L-Ascorbic acid, 6-octadecanoate, (21.41) Octacosane, (21.524) Erucic acid and (22.71) Triacontane

P/EA	(10.816) Hexamethylbenzene, (11.512) Butylated Hydroxytoluene, (11.902) Oleic Acid, (12.786) Phytol, (13.164) Methyl tetradecanoate, (13.377) 6-tert-Butyl-2,4-dimethylphenol, (13.974) Palmitaldehyde, (14.109) Levomenthol, (14.24) Isophytol, (14.36) Hexadecanoic acid & methyl ester, (14.884) Isopropyl palmitate, (15.407) Linolenic acid & methyl ester, (15.772) Methyl stearate, (16.085) Isopropyl linoleate, (16.277) Tetracosanoic acid, (17.569) 2-Hexadecoxyethanol, (17.668) Methyl tridecanoate, (18.23) Arachic acid, (18.779) Salsoline, (20.25) Hexacosane, (20.898) L-Ascorbic acid & 6-octadecanoate, (21.411) Octacosane and (22.715) Triacontane

O/DEE	(6.567) *β*-Hydroxydodecanoic acid, (7.038) Linoleic acid, (7.607) Hexadecenoic acid & Z-11, (7.699) 2-Hexadecanol, (8.014) Hexadecanedicarboxylic acid, (9.008) Erucic acid, (9.429) Benzyl laurate, (9.606) Thymol, (9.716) Cuminic alcohol, (9.853) Methyl phytanate, (10.38) Retinol, (10.809) Hexamethylbenzene, (11.502) Butylated Hydroxytoluene, (11.9) Oleic Acid, (12.729) Phytol, (13.16) Methyl tetradecanoate, (13.374) 6-tert-Butyl-2,4-dimethylphenol, (13.971) Palmitaldehyde, (14.116) Levomenthol, (14.24) Isophytol, (14.362) Hexadecanoic acid & methyl ester, (14.83) Isopropyl palmitate, (15.407) Linolenic acid & methyl ester, (15.77) Methyl stearate, (16.086) Isopropyl linoleate, (16.277) Tetracosanoic acid, (17.568) 2-Hexadecoxyethanol, (17.668) Methyl tridecanoate, (18.245) Arachic acid, (18.779) Salsoline, (20.25) Hexacosane, (20.865) L-Ascorbic acid & 6-octadecanoate, (21.41) Octacosane and (22.73) Triacontane.

O/EA	(10.38) Retinol, (11.502) Butylated Hydroxytoluene, (12.167) 1-Hexadecanol, (13.164) Methyl tetradecanoate, (13.943) Palmitaldehyde, (14.109) Levomenthol, (14.215) Isophytol, (14.399) Hexadecanoic acid & methyl ester, (14.832) Isopropyl palmitate, (15.407) Linolenic acid & methyl ester, (15.771) Methyl stearate, (16.083) Isopropyl linoleate, (20.25) Hexacosane and (22.718) Triacontane.

## Data Availability

All data used to support the findings of this study are included within the article and the supplementary information files.

## Supplementary Materials

Supplementary 1 Table S1: the abbreviation of each extract according to the* Moringa* species leaves and the solvents used (docx).

Supplementary 2 Table S2: primers sequence used for the tested genes (docx).

Supplementary 3 Fig S1: schematic diagram of the extraction procedures (TIF).
