# Supplementary material for: Moringa peregrina Leaves Extracts Induce Apoptosis and Cell Cycle Arrest of Hepatocellular Carcinoma
Source: Biomed Res Int. 2019 Jan 1;2019:2698570. doi: 10.1155/2019/2698570 (PMC6332967; doi:10.1155/2019/2698570)
Supplement: Supplementary 1 — Table S1: the abbreviation of each extract according to the Moringa species leaves and the solvents used (docx). [file 2698570.f1.docx]

**S1 Table.** The abbreviation of each extract according to the Moringa species leaves and the solvents used.

|  | Hexane | Diethyl Ether | Ethyl Acetate | Methanol | Acetonitrile |
| --- | --- | --- | --- | --- | --- |
| *Moringa peregrina* leaves | P/H | P/DEE | P/EA | P/MeOH | P/ACN |
| *Moringa oleifera* leaves | O/H | O/DEE | O/EA | O/MeOH | O/ACN |
