# Supplementary material for: Moringa peregrina Leaves Extracts Induce Apoptosis and Cell Cycle Arrest of Hepatocellular Carcinoma
Source: Biomed Res Int. 2019 Jan 1;2019:2698570. doi: 10.1155/2019/2698570 (PMC6332967; doi:10.1155/2019/2698570)
Supplement: Supplementary 2 — Table S2: primers sequence used for the tested genes (docx). [file 2698570.f2.docx]

**S2 Table.** Primers sequence used for the tested genes.

| Genes | Primer sequence |
| --- | --- |
| *BAX* | Forward: 5′-TTC CGA GTG GCA GCT GAG ATG TTT-3′ |
|  | Reverse: 5′-TGC TGG CAA AGT AGA AGA GGG CAA-3′ |
| *BCL2* | Forward: 5′-CAT GCC AAG AGG GAA ACA CCA GAA-3′ |
|  | Reverse: 5′-GTG CTT TGC ATT CTT GGA TGA GGG-3′ |
| *P53* | Forward: 5′-GCC CAA CAA CAC CAG CTC CT -3′ |
|  | Reverse: 5′-CCT GGG CAT CCT TGA GTT CC-3′ |
| *CASP3* | Forward: 5′-TTC ATT ATT CAG GCC TGC CGA GG-3′ |
|  | Reverse: 5′-TTC TGA CAG GCC ATG TCA TCC TCA-3′ |
| *MMP1* | Forward: 5′-CTG GCC ACA ACT GCC AAA TG-3′ |
|  | Reverse: 5′-CTG TCC CTG AAC AGC CCA GTA CTT A-3′ |
| *GAPDH* | Forward: 5′-TTC CAG GAC CAA GAT CCC TCC AAA-3′ |
|  | Reverse: 5′-ATG GTG GTG AAG ACA CCA GTG AAC-3′ |
