# Supplementary material for: Moringa peregrina Leaves Extracts Induce Apoptosis and Cell Cycle Arrest of Hepatocellular Carcinoma
Source: Biomed Res Int. 2019 Jan 1;2019:2698570. doi: 10.1155/2019/2698570 (PMC6332967; doi:10.1155/2019/2698570)
Supplement: Supplementary 3 — Fig S1: schematic diagram of the extraction procedures (TIF). [file 2698570.f3.pptx]

## Slide 1
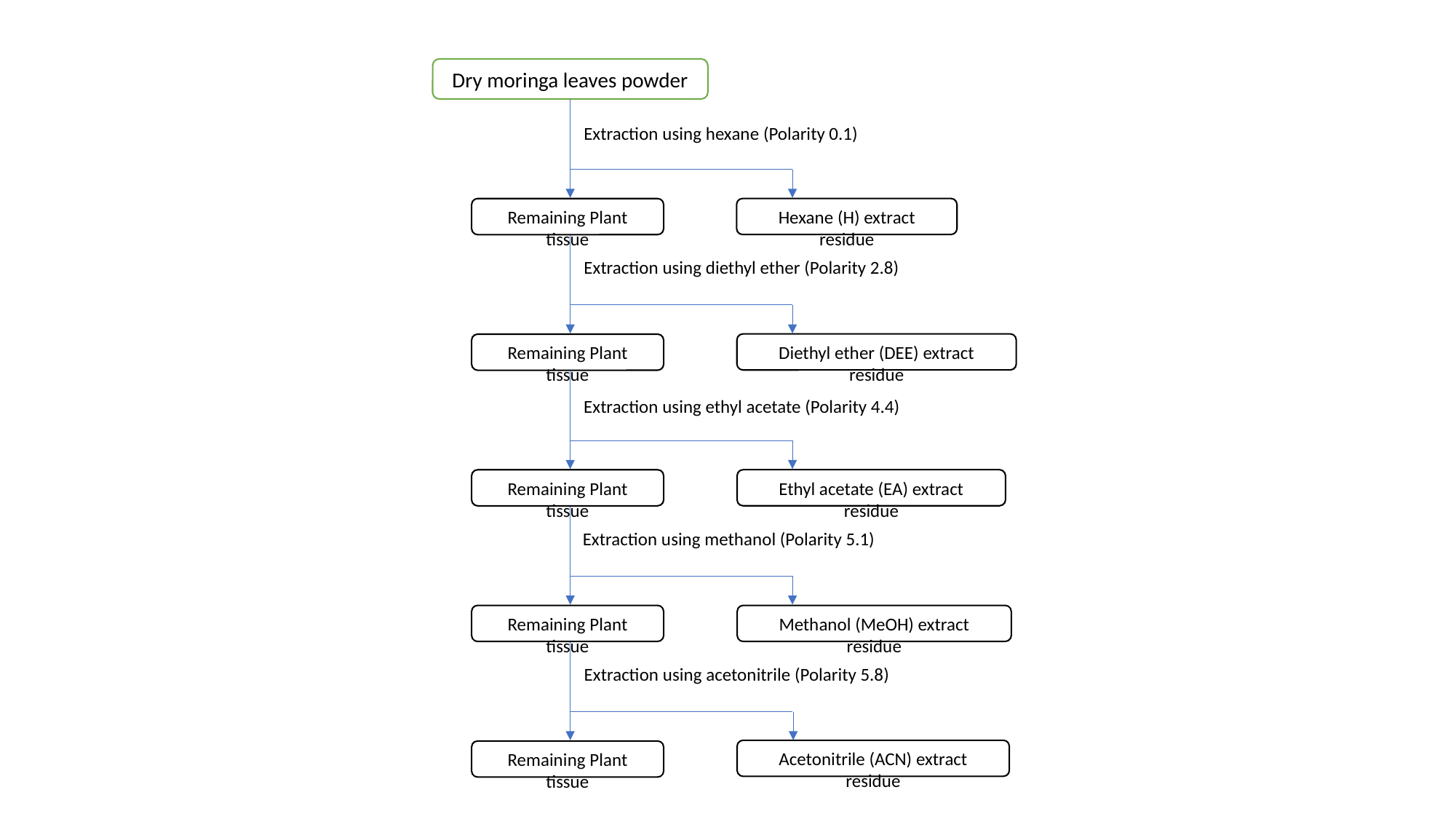

Dry moringa leaves powder
Extraction using hexane (Polarity 0.1)
Hexane (H) extract residue
Remaining Plant tissue
Extraction using diethyl ether (Polarity 2.8)
Diethyl ether (DEE) extract residue
Remaining Plant tissue
Extraction using ethyl acetate (Polarity 4.4)
Ethyl acetate (EA) extract residue
Remaining Plant tissue
Extraction using methanol (Polarity 5.1)
Methanol (MeOH) extract residue
Remaining Plant tissue
Extraction using acetonitrile (Polarity 5.8)
Acetonitrile (ACN) extract residue
Remaining Plant tissue
